# Diaphragmatic Pleurisy

**Published:** 1907-05-18

**Authors:** Leonard J. Kidd

**Affiliations:** 35a Welbeck Street, W.


### EDITOR'S LETTER-BOX.

[Our Correspondents are reminded that prolixity is a great bar tc publication, and that brevity of style and conciseness of statement greatly facilitate early insertion.]

### DIAPHRAGMATIC PLEURISY.

SIR,—Dr. J. A. Nixon, in his paper in your issue of May 11, 1907, omits to mention as one of the symptoms a fixed severe pain in one or both shoulders over the fourth cervical area. This is not very uncommon : is almost always overlooked, because not one medical man in 500 probably knows of it. I have seen it myself, and have known it to be overlooked.

So far back as 1890 (I write from memory) Dr. John Ferguson wrote a short paper in *Brain* on "Sensory Fibres in the Phrasic Nerve." In it he detailed the result of some experiments he made in cats. In Dr. Henry Head's famous papers in *Brain* some few years later he referred (very briefly and inadequately, however) to shoulder pain in connection with the diaphragm.

In December 1896 a very near medical relative of mine wrote to me about his wife who, recently confined, had febrile symptoms and a fixed severe pain in one shoulder that had completely puzzled him and another medical relative of ours. Knowing of the shoulder pain of diaphragmatic pleurisy, and knowing further that the patient had had pleuro-pneumonia, I wrote and told him of the connection between shoulder pain and lesions of the diaphragmatic pleura, and advised a blister over the region of the diaphragm on the affected side. He replied it was too late for my blister (clearly the trouble had resolved meanwhile). I remember that soon after I read Dr. Ferguson's paper in 1890, I asked my brother, Dr. Percy Kidd, if he had ever seen shoulder pain in diaphragmatic pleurisy. He said he had once or twice.

I admit the connection is probably not very common, but I do say that it is always almost overlooked because the anatomical connection between the phrenic and the acromial branches of the cervical plexus is forgotten. The result is the chest is not examined; further, as these conditions almost certainly often get well of themselves, the connection is almost certainly much commoner than is believed.

I am certain that in those cases of habit-spasm that show hiccup and grunting spasmodic noises there is often underlying them old diaphragmatic pleuro-pneumonia or pleurisy. I pointed this out to a famous neurologist one day in the Queen Square Hospital; it was evident the matter was new to him, and he was sensible enough to see at once what a good point it was.

I am, Sir, yours faithfully,

LEONARD J. KIDD, M.D.

35A Welbeck Street, W.

THE HOSPITAL *May* 18, 1907.

